# High expression of PARD3 predicts poor prognosis in hepatocellular carcinoma

**DOI:** 10.1038/s41598-021-90507-w

**Published:** 2021-05-26

**Authors:** Songwei Li, Jian Huang, Fan Yang, Haiping Zeng, Yuyun Tong, Kejia Li

**Affiliations:** 1 Department of Interventional Radiology, The Second Affiliated Hospital of Kunming Medical University, 374 Dianmian Avenue, Kunming, 650101 Yunnan China; 2 Department of Pharmacy, The Second Affiliated Hospital of Kunming Medical University, 374 Dianmian Avenue, Kunming, 650101 Yunnan China; 3 Department of Infection Management, The Second Affiliated Hospital of Kunming Medical University, 374 Dianmian Avenue, Kunming, 650101 Yunnan China

**Keywords:** Cancer, Computational biology and bioinformatics, Biomarkers, Gastroenterology, Oncology

## Abstract

Hepatocellular carcinoma (HCC) is one of the most commonly cancers with poor prognosis and drug response. Identifying accurate therapeutic targets would facilitate precision treatment and prolong survival for HCC. In this study, we analyzed liver hepatocellular carcinoma (LIHC) RNA sequencing (RNA-seq) data from The Cancer Genome Atlas (TCGA), and identified PARD3 as one of the most significantly differentially expressed genes (DEGs). Then, we investigated the relationship between PARD3 and outcomes of HCC, and assessed predictive capacity. Moreover, we performed functional enrichment and immune infiltration analysis to evaluate functional networks related to PARD3 in HCC and explore its role in tumor immunity. PARD3 expression levels in 371 HCC tissues were dramatically higher than those in 50 paired adjacent liver tissues (*p* < 0.001). High PARD3 expression was associated with poor clinicopathologic feathers, such as advanced pathologic stage (*p* = 0.002), vascular invasion (*p* = 0.012) and TP53 mutation (*p* = 0.009). Elevated PARD3 expression also correlated with lower overall survival (OS, HR = 2.08, 95% CI = 1.45–2.98, *p* < 0.001) and disease-specific survival (DSS, HR = 2.00, 95% CI = 1.27–3.16, *p* = 0.003). 242 up-regulated and 71 down-regulated genes showed significant association with PARD3 expression, which were involved in genomic instability, response to metal ions, and metabolisms. PARD3 is involved in diverse immune infiltration levels in HCC, especially negatively related to dendritic cells (DCs), cytotoxic cells, and plasmacytoid dendritic cells (pDCs). Altogether, PARD3 could be a potential prognostic biomarker and therapeutic target of HCC.

## Introduction

Hepatocellular carcinoma (HCC) is the fifth most commonly diagnosed cancer, accounting for 7% of all cancers worldwide^1^, and furthermore, the incidence of HCC continues increasing by 2–3% annually^2,3^. With a 5-year overall survival of 12–18%, HCC ranks second in terms of cancer-related death^1,2,4,5^. In the past decades, substantial progress has been made in prevention, diagnosis, and treatment for HCC^6–8^. However, due to insidious onset, rapid progression and a lack of effective screening strategies, less than 30% of HCC patients can be diagnosed at an early stage, and have the opportunity to undergo radical treatments^4^. Transarterial chemoembolization or systemic therapies are widely recommended for patients with advanced disease^4,5^, but unfortunately, the improvement in prognosis is not satisfactory enough even with the latest targeted drugs or immune-based therapies^9–13^. Therefore, identifying more accurate prognostic biomarkers and therapeutic targets would facilitate precision treatment and prolong survival for HCC patients.

Cell polarity is essential for epithelial cells to maintain normal morphology and perform physiological functions^14^. Aberrant cell polarity, a hallmark of cancers, is implicated in tumor formation, growth, invasion, and metastasis^15^. Cell polarity is regulated by sets of evolutionarily conserved polarity proteins including the partitioning-defective (Par) complex, Scribble complexes, and Crumbs complexes^16^. The Par complex, which has the most ubiquitous function among these proteins, consists of Par3, Par6, and atypical protein kinase C (aPKC)^17^. Par3 serves as an adaptor protein for the assembly of Par complex and multiple proteins, such as the Rac-GEF, Tiam1 or Rho GTPases, thereby activating polarity signaling^16,18^. PARD3, encoding Par3 protein, is a single-copy gene with 26 exons, and located on chromosome 10p11.22-p11.21^17^. Deleterious variants of PARD3 were first detected in neural tube defects, coeliac disease and ulcerative colitis^19–21^, and subsequently, a series of studies identified the dual function of PARD3 in different malignant tumors of epithelial origin^14–18,22–27^. However, the specific role and detailed mechanism of PARD3 in HCC has not been fully elucidated^17^.

To screen a biomarker closely related to the formation and progression of HCC, we analyzed the differential expression of PARD3 and its clinicopathological relevance, using liver hepatocellular carcinoma (LIHC) RNA sequencing (RNA-seq) data of HCC patients from The Cancer Genome Atlas (TCGA). Then, we put PARD3 into a prognosis analysis in order to evaluate predictive capacity. Moreover, we performed comparative transcriptome analysis, functional enrichment analysis and correlation analysis between PARD3 and immune cell infiltration to evaluate functional networks related to PARD3 in HCC and explore its role in tumor immunity.

## Results

### Overexpression of PARD3 in HCC

**Figure 1:**
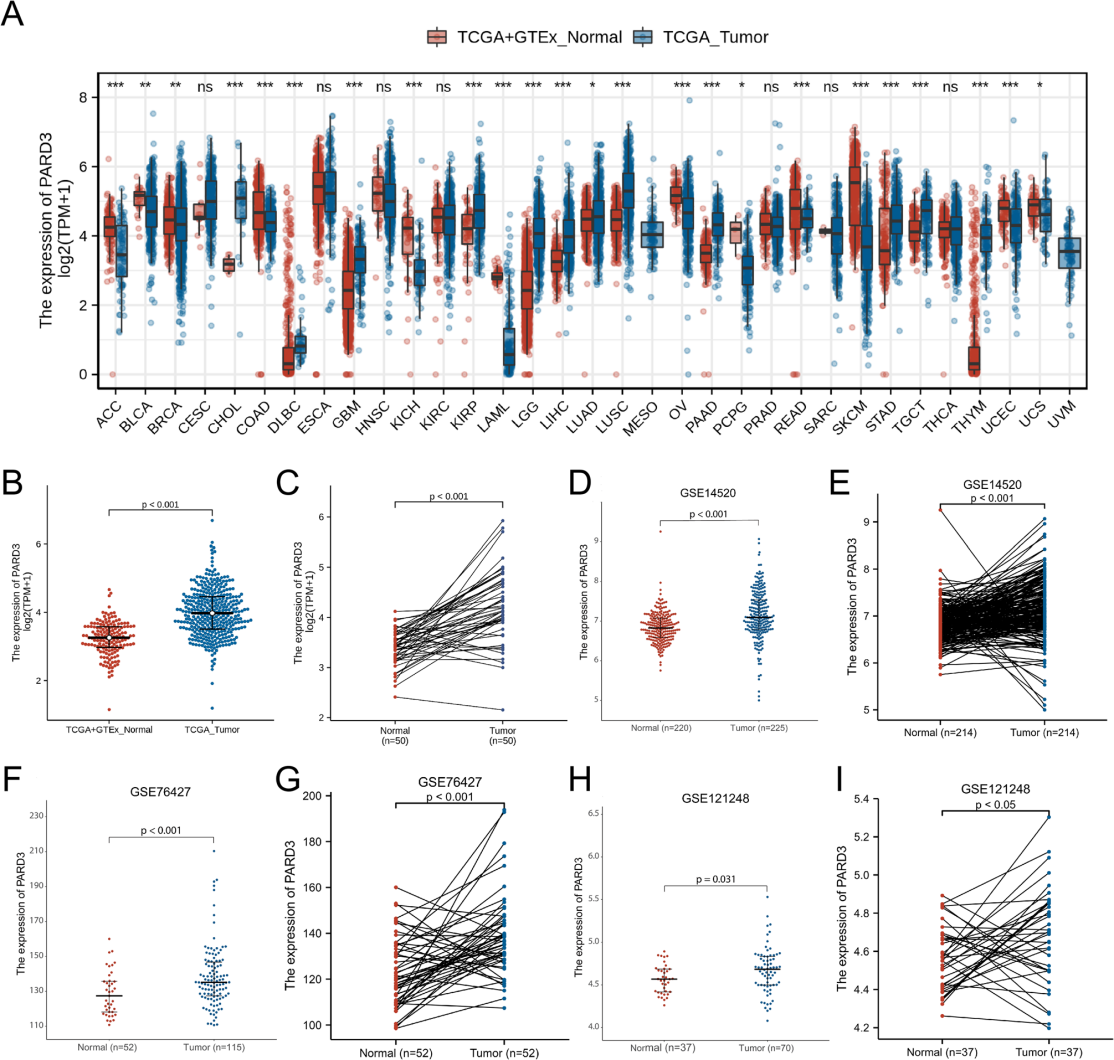
Differential expression of PARD3. (**A**) Differential expression of PARD3 in 33 types of human cancers and normal tissues from TCGA and GTEx (*p < 0.05, **p < 0.01, ***p < 0.001, ns: *p* ≥ 0.05). (**B**) Differential expression of PARD3 in HCC and normal liver tissues from TCGA and GTEx. (**C**) Differential expression of PARD3 in paired HCC and their adjacent liver tissues from TCGA. (**D**,**F**,**H**) Differential expression of PARD3 in HCC and normal liver tissues from GSE14520, GSE76427 and GSE121248. (**E**,**G**,**I**) Differential expression of PARD3 in paired HCC and their adjacent liver tissues from GSE14520, GSE76427 and GSE121248.

We initially compared PARD3 expression between tumor and normal tissues in multiple cancer types using the UCSC Xena database. As shown in Fig. 1A, PARD3 expression was significantly higher in cholangiocarcinoma (CHOL), Diffuse large B-cell lymphoma (DLBC), glioblastoma (GBM), kidney renal papillary cell carcinoma (KIRP), low-grade glioma (LGG), liver hepatocellular carcinoma (LIHC), lung adenocarcinoma (LUAD), lung squamous cell carcinoma (LUSC), pancreatic adenocarcinoma (PAAD), stomach adenocarcinoma (STAD), testicular germ cell tumor (TGCT) and thymic carcinoma (THYM). In contrast, PARD3 expression was significantly lower in adrenocortical carcinoma (ACC), bladder urothelial carcinoma (BLCA), breast invasive carcinoma (BRCA), colon adenocarcinoma (COAD), kidney chromophobe (KICH), acute myeloid leukemia (LAML), ovarian serous cystadenocarcinoma (OV), pheochromocytoma and paraganglioma (PCPG), rectum adenocarcinoma (READ), skin cutaneous melanoma (SKCM), uterine corpus endometrial carcinoma (UCEC) and uterine carcinosarcoma (UCS).

Then, we focused on PARD3 expression in HCC patients. The data from TCGA and GTEx revealed that PARD3 expression levels in 371 HCC tissues were dramatically higher than those in normal liver tissues, which was validated in 50 paired HCC and their adjacent liver tissues (Fig. 1B,C). The data from other three independent cohorts (GSE14520, GSE76427 and GSE121248) confirmed the above results that PARD3 was overexpressed in HCC patients (Fig. 1D–I).

### Correlation of PARD3 overexpression with poor clinicopathologic features

**Figure 2:**
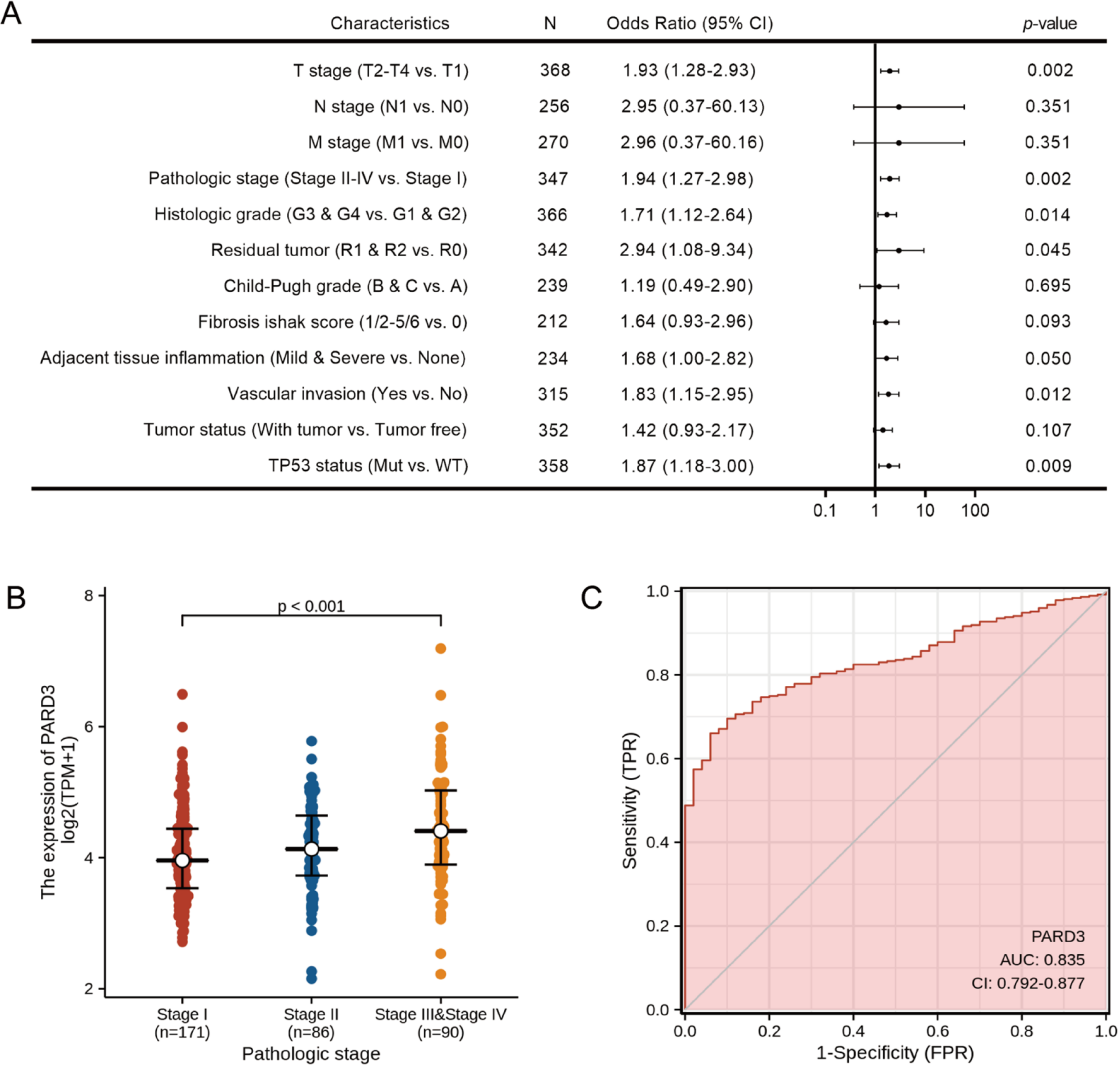
Correlation between PARD3 expression and clinicopathologic characteristics in HCC. (**A**) Correlation between PARD3 expression (categorical dependent variable) and clinicopathologic characteristics (logistic regression; Odds Ratio equals to the ratio of odds_high expression_ to odds_low expression_). (**B**) High PARD3 expression (categorical dependent variable) was associated with advanced pathologic stages. (**C**) Time-dependent receiver operation characteristic (ROC) curve of PARD3. False positive rate (FPR) is indicated on the abscissa; and true positive rate (TPR) is indicated on the ordinate.

To investigate overexpression of PARD3 and its clinicopathological relevance, 371 HCC samples with detailed patient information (retrieved from TCGA in June 2020) were divided into two groups by the median value of PARD3 expression. As shown in Table 1, high PARD3 expression was associated with advanced T stage, pathologic stage, residual tumor, histologic grade, vascular invasion and higher alpha fetoprotein (AFP). Otherwise, high PARD3 expression group also carried more TP53 mutation (Mut) than low PARD3 expression group. Whereas, the distributions of other clinicopathologic features showed no difference between high and low PARD3 expression group. Univariate logistic regression further confirmed the association between high PARD3 expression and poor clinicopathologic characteristics in HCC patients (Fig. 2A,B). In addition, the area under receiver operation characteristic (ROC) curve (AUC, AUC = 0.835, 95% CI = 0.792–0.877) indicated that PARD3 had a good diagnostic power, and was expected to be a potential biomarker for HCC (Fig. 2C).

**Table 1:** Clinicopathologic characteristics in LIHC cohort according to PARD3 expression.

Characteristics	level	Low expression of PARD3	High expression of PARD3	*p*-value
n		186	185	
Gender (%)	Female	56 (30.1%)	65 (35.1%)	0.356
	Male	130 (69.9%)	120 (64.9%)	
Race (%)	Asian	78 (44.3%)	80 (43.7%)	0.983
	Black or African	8 (4.5%)	9 (4.9%)	
	White	90 (51.1%)	94 (51.4%)	
T stage (%)	T1	105 (57.4%)	76 (41.1%)	0.006^†^
	T2	43 (23.5%)	51 (27.6%)	
	T3	32 (17.5%)	48 (25.9%)	
	T4	3 (1.6%)	10 (5.4%)	
N stage (%)	N0	125 (99.2%)	127 (97.7%)	0.622^‡^
	N1	1 (0.8%)	3 (2.3%)	
M stage (%)	M0	132 (99.2%)	134 (97.8%)	0.622^‡^
	M1	1 (0.8%)	3 (2.2%)	
Pathologic stage (%)	Stage I	100 (57.5%)	71 (41.0%)	0.006^†,‡^
	Stage II	41 (23.6%)	45 (26.0%)	
	Stage III	31 (17.8%)	54 (31.2%)	
	Stage IV	2 (1.1%)	3 (1.7%)	
Tumor status (%)	Tumor free	108 (61.4%)	93 (52.8%)	0.132
	With tumor	68 (38.6%)	83 (47.2%)	
Residual tumor (%)	R0	172 (97.2%)	152 (92.1%)	0.023^†,‡^
	R1	4 (2.3%)	13 (7.9%)	
	R2	1 (0.6%)	0 (0.0%)	
Histologic grade (%)	G1	36 (19.6%)	19 (10.4%)	0.023^†^
	G2	92 (50.0%)	85 (46.7%)	
	G3	52 (28.3%)	70 (38.5%)	
	G4	4 (2.2%)	8 (4.4%)	
Adjacent hepatic tissue inflammation (%)	Mild	46 (37.4%)	53 (47.7%)	0.144
	None	69 (56.1%)	48 (43.2%)	
	Severe	8 (6.5%)	10 (9.0%)	
Child–Pugh grade (%)	A	118 (91.5%)	99 (90.0%)	0.810^‡^
	B	10 (7.8%)	11 (10.0%)	
	C	1 (0.8%)	0 (0.0%)	
Fibrosis Ishak score (%)	0	47 (39.8%)	27 (28.7%)	0.405
	1–2	16 (13.6%)	15 (16.0%)	
	3–4	15 (12.7%)	13 (13.8%)	
	5–6	40 (33.9%)	39 (41.5%)	
Vascular invasion (%)	No	116 (72.0%)	90 (58.4%)	0.016^†^
	Yes	45 (28.0%)	64 (41.6%)	
TP53 status (%)	Mut	40 (22.2%)	62 (34.8%)	0.012^†^
	WT	140 (77.8%)	116 (65.2%)	
Age (median [IQR])		61.50 [53.00, 69.00]	60.50 [51.00, 69.00]	0.329
Height (median [IQR])		168.00 [161.50, 174.00]	168.00 [161.00, 173.00]	0.590
Weight (median [IQR])		72.00 [60.00, 84.00]	68.00 [59.00, 81.00]	0.210
BMI (median [IQR])		24.84 [22.14, 28.88]	24.19 [21.24, 28.43]	0.256
AFP (ng/ml) (median [IQR])		10.00 [3.00, 52.00]	28.00 [5.00, 1456.00]	0.001^†^
Alb (g/dl) (median [IQR])		4.00 [3.42, 4.40]	4.00 [3.55, 4.30]	0.520
PT (s) (median [IQR])		1.10 [1.00, 8.93]	1.10 [1.00, 9.57]	0.538

*Mut* mutant, *WT* wild type, *BMI* body mass index, *AFP* alpha fetoprotein, *Alp* albumin, *TB* total bilirubin, *PT* prothrombin time.

^†^Statistically significant.

^‡^Fisher exact test.

### Correlation of PARD3 overexpression with adverse Outcomes in HCC

**Figure 3:**
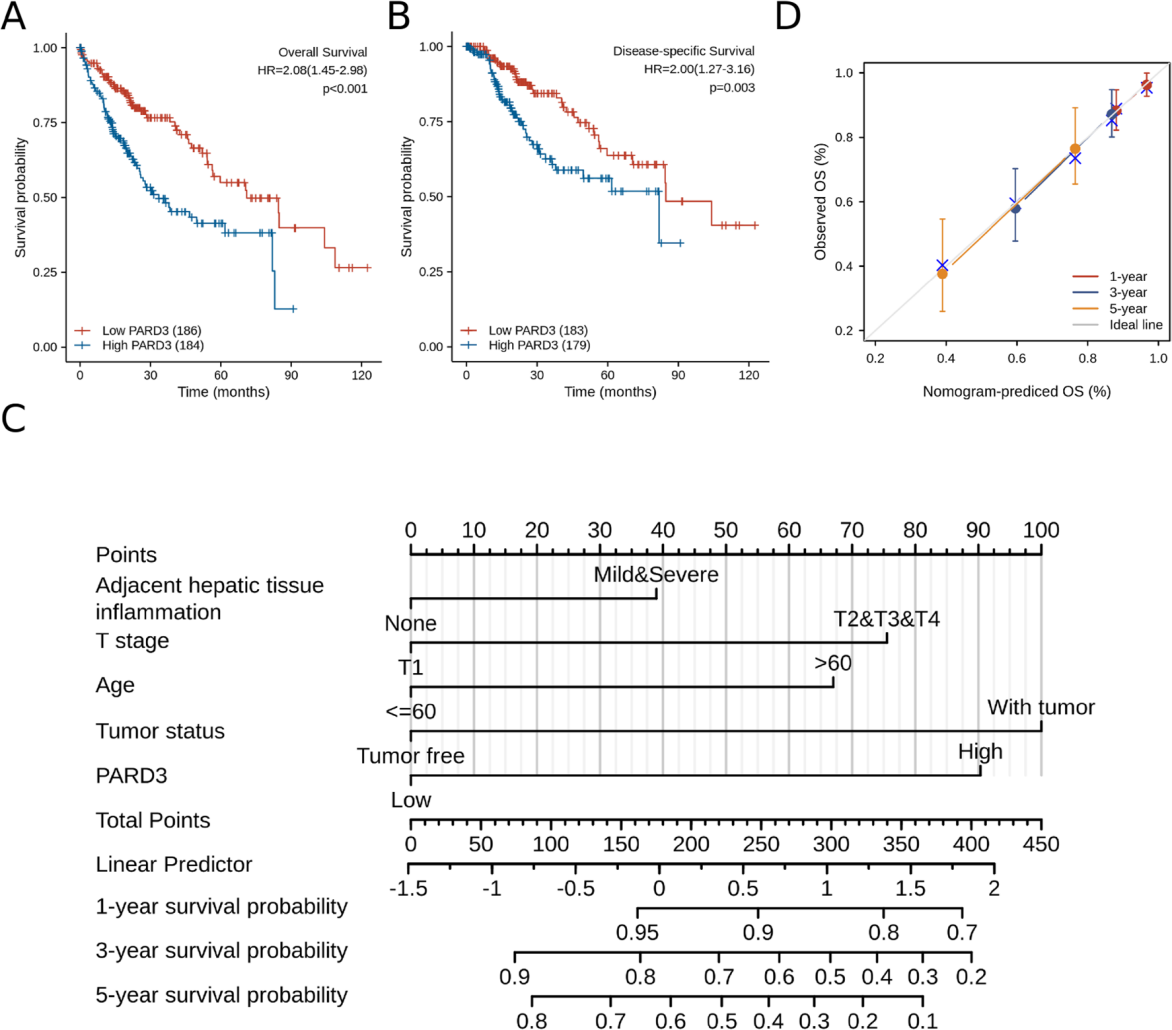
Impact of PARD3 expression on survival in HCC patients. (**A**) Overall survival (OS) was significantly higher in low PARD3 expression group (HR = 2.08, 95% CI = 1.45–2.98, *p* < 0.001); (**B**) Disease-specific survival (DSS) was significantly higher in low PARD3 expression group (HR = 2.00, 95% CI = 1.27–3.16, *p* = 0.003); (**C**) Nomogram for prediction of OS in HCC patients (existing adjacent hepatic tissue inflammation, T2–T4 stage, age > 60 years, with tumor, and high PARD3 expression were converted into 39, 75.5, 67, 100 and 90.25 points, respectively. The total points accumulated by the above covariates correspond to the predicted probability for an HCC patient); (**D**) Calibration curve of the nomogram.

As PARD3 overexpression was correlated with poor clinicopathologic features, we then explored the prognostic value of PARD3 in HCC. Kaplan–Meier survival curve demonstrated that elevated PARD3 expression led to decreased overall survival (OS, HR = 2.08, 95% CI = 1.45–2.98, *p* < 0.001) and disease-specific survival (DSS, HR = 2.00, 95% CI = 1.27–3.16, *p* = 0.003) (Fig. 3A,B), which was validated through TISIDB database and other two independent cohorts in GEO database (GSE76427 and GSE14520) (Fig. S1). Further univariate and multivariate Cox analysis showed that PARD3 was an independent risk factor for HCC patients leading to adverse outcomes, and tumor status was the other negative factor (Table S1 and S2)^28^.

Based on multivariate COX regression, we constructed a nomogram to provide a visual, intuitive and quantitative description of those risk factors and their weights in HCC, and predict the probability of 1-year, 3-year and 5-year survival. Potential covariates included adjacent hepatic tissue inflammation (39 points), T stage (75.5 points), age (67 points), tumor status (100 points) and PARD3 expression (90.25 points), and higher total points indicated worse prognosis (Fig. 3C).

Then, we made calibration curve of the nomogram, and calculated concordance index (C-index) to assess the predictive ability of PARD3 as a biomarker for HCC. As shown in Fig. 3D, the bias-corrected line in the calibration curve was close to the ideal line, and the C-index was 0.702 (95% CI = 0.668–0.736). In all, the nomogram was available to predict the prognosis of HCC patients, and PARD3 exhibited stable predictive ability.

### Co-expression genes and biological functions related to PARD3 in HCC

**Figure 4:**
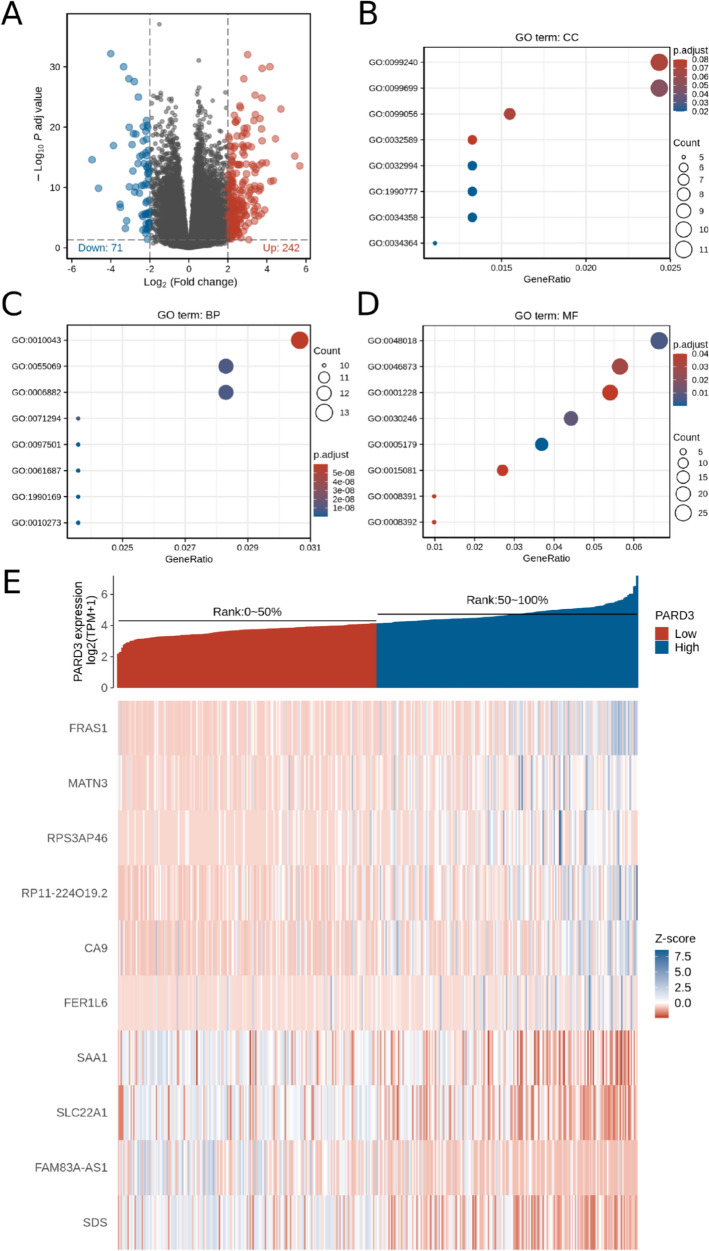
Co-expression genes and biological functions related to PARD3 in HCC. (**A**) Volcano plot of differential gene profiles between high and low PARD3 expression groups (|logFC| > 2 and adjusted *p*-value < 0.05); (**B**) Cellular components (CCs) enrichment; (**C**) Biological processes (BPs) enrichment; (**D**) Molecular functions (MFs) enrichment; (**E**) Expression heatmap of top 10 PARD3-associated genes. Description of GO identifiers: GO:0034358: plasma lipoprotein particle; GO:1990777: lipoprotein particle; GO:0032994: protein-lipid complex; GO:0034364: high-density lipoprotein particle; GO:0099699: integral component of synaptic membrane; GO:0099056: integral component of presynaptic membrane; GO:0099240: intrinsic component of synaptic membrane; GO:0032589: neuron projection membrane. GO:0010273: detoxification of copper ion; GO:1990169: stress response to copper ion; GO:0061687: detoxification of inorganic compound; GO:0097501: stress response to metal ion; GO:0006882: cellular zinc ion homeostasis; GO:0055069: zinc ion homeostasis; GO:0071294: cellular response to zinc ion; GO:0010043: response to zinc ion. GO:0005179: hormone activity; GO:0048018: receptor ligand activity; GO:0030246: carbohydrate binding; GO:0046873: metal ion transmembrane transporter activity; GO:0008392: arachidonic acid epoxygenase activity; GO:0008391: arachidonic acid monooxygenase activity; GO:0001228: DNA-binding transcription activator activity, RNA polymerase II-specific; GO:0015081: sodium ion transmembrane transporter activity.

To overview the biological roles of PARD3 in HCC, we conducted gene ontology (GO) enrichment analysis of PARD3 and its associated identify differentially expressed genes (DEGs), including biological processes (BPs), molecular functions (MFs) and cellular components (CCs). As illustrated in the volcano plot (Fig. 4A), 242 up-regulated and 71 down-regulated genes were significantly related with PARD3 expression, top 10 of which were presented in the heatmap (Fig. 4E). As the Par complex consists of Par3, Par6, and aPKC, we specifically analyzed the differentially expressing of PARD6A/B/G, encoding Par6, and PRKCI/Z, encoding aPKC using Spearman correlation test. The correlation values of PARD6A, PARD6B, PARD6G, PRKCI and PRKCIZ were − 0.01 (*p* > 0.05), 0.51 (*p* < 0.001), 0.54 (*p* < 0.001), 0.64 (*p* < 0.001) and 0.23 (*p* < 0.001), respectively (Table S3).

The result of CCs indicated that PARD3 and its associated DEGs were primarily located in lipoprotein particles, synaptic membranes and neuron projection membranes (Fig. 4B). In terms of BPs and MFs, these genes mainly participated in detoxification of copper ion (Cu), stress response to metal ions, and zinc ion (Zn) homeostasis, which associated with receptor ligand activity, metal ion transmembrane transporter activity, DNA-binding transcription activator activity, hormone activity, carbohydrate binding, and arachidonic acid oxygenase activity (Fig. 4C,D).

**Figure 5:**
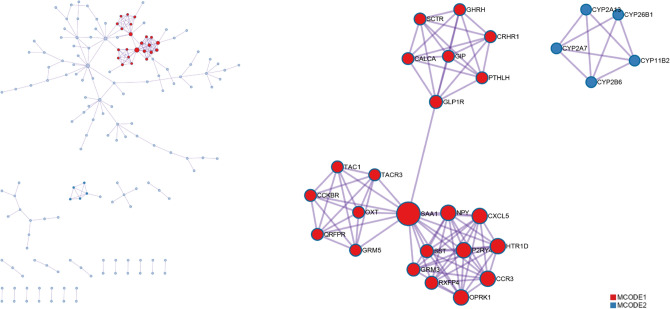
Protein–protein interaction (PPI) network of PARD3-associated pathways.

Signaling pathways, working as functional units of gene groups, play an important role in cell biological effects. Thus, we identified significantly enriched signaling pathways between low and high PARD3 expression groups by Gene set enrichment analysis (GSEA). According to normalized enrichment scores (NSEs), 431 pathways were significantly associated with high expression of PARD3, including cell cycle and mitosis, DNA double strand break repair, cell motility, Rho, MAPK, TP53, response to metal ions, and pathways related to metabolism (Table 2). In addition, we made a protein–protein interaction (PPI) to highlight the most important protein functional groups interacting with each other. The two most crucial MCODE subnetworks were SAA1-related cluster and CYP-related cluster, both of which were involved in metabolism and homeostasis (Fig. 5). The above result suggested a negative impact of PARD3 on tumorigenesis and progression of HCC.

**Table 2:** Pathways enriched in high expression groups using GSEA.

Name	NES	Adjusted *p*-value	FDR *q*-value
REACTOME_CELL_CYCLE_MITOTIC	2.718	0.019	0.013
REACTOME_RHO_GTPASES_ACTIVATE_FORMINS	2.653	0.019	0.013
REACTOME_FCERI_MEDIATED_MAPK_ACTIVATION	2.468	0.019	0.013
REACTOME_DNA_DOUBLE_STRAND_BREAK_REPAIR	2.399	0.019	0.013
REACTOME_MET_PROMOTES_CELL_MOTILITY	2.314	0.019	0.013
REACTOME_REGULATION_OF_TP53_ACTIVITY	2.111	0.019	0.013
REACTOME_PYRIMIDINE_CATABOLISM	− 2.090	0.019	0.013
REACTOME_RESPONSE_TO_METAL_IONS	− 2.608	0.019	0.013
KEGG_FATTY_ACID_METABOLISM	− 2.575	0.019	0.013
KEGG_GLYCINE_SERINE_AND_THREONINE_METABOLISM	− 2.727	0.019	0.013

*FDR* false discovery rate, *NES* normalized enrichment score.

### Association of PARD3 with immune infiltration in HCC

**Figure 6:**
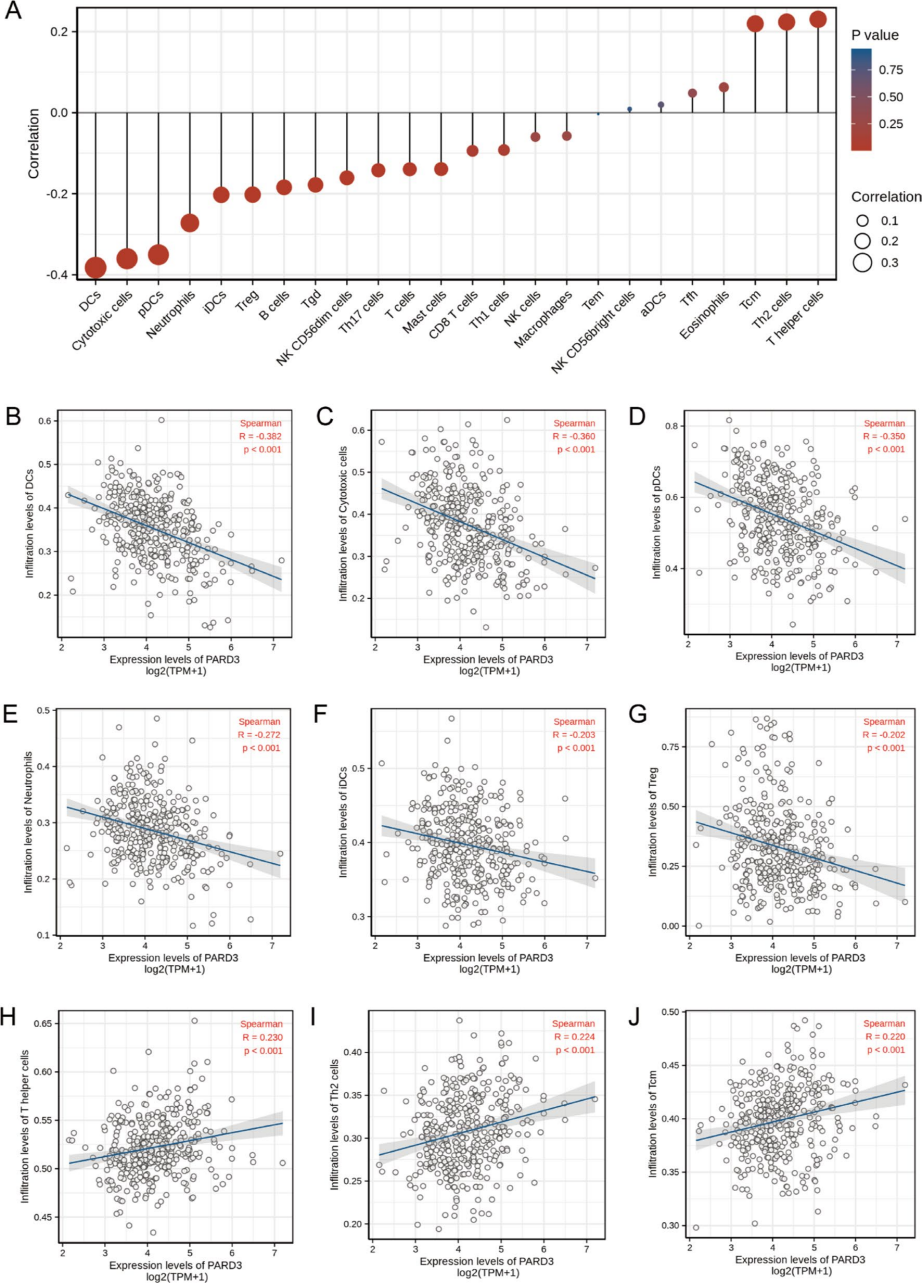
Association of PARD3 with immune infiltration. (**A**) Association of PARD3 with immune infiltration (*DCs* dendritic cells, *pDCs* plasmacytoid DCs, *iDCs* immature DCs, *aDCs* activated DCs, *Treg* regulatory T cells, *Tgd* T gamma delta, *Th* helper T cells, *Tfh* T follicular helper, *NK* natural killer, *Tem* T effector memory, *Tcm* T central memory). (**B**–**G**) PARD3 was negatively related to infiltration of DCs, cytotoxic cells, pDCs, neutrophils, iDCs, and Treg (*p* < 0.01). (**H**–**J**) PARD3 was positively related to in infiltration of T helper cells, Th2 cells, and Tcm (*p* < 0.01).

Immune infiltration, which influences tumor purity, is one of the major risk factors in cancers^29–31^, hence we quantified the enrichment scores (ECs) of 24 types of tumor-infiltrating immune cells (TIICs), in order to evaluate the association between PARD3 and immune infiltration levels in HCC. As illustrated in Fig. 6, PARD3 was involved in infiltration of T helper cells, Th2 cells, and T central memory (Tcm); but negatively related to infiltration of dendritic cells (DCs), cytotoxic cells, plasmacytoid dendritic cells (pDCs), neutrophils, immature DCs (iDCs), and regulatory T cells (Treg). Furthermore, we replicated immune infiltration analysis using another tumor-immune system interaction database (TISIDB), and obtained consistent results (Fig. S2).

## Discussion

PARD3 plays a crucial role in establishment and maintenance of epithelial cell polarity^23^. So far, at least five PARD3 variants have been identified in human liver cDNA library^32^. PARD3 largely engages in cancer cell proliferation, apoptosis, invasion, migration and epithelial-mesenchymal transition (EMT)^18,24,33,34^. The modulation of PARD3 in tumorigenesis and progression among different cancers seems to be controversial. For instance, PARD3 acts as a tumor suppressor in lung, bladder, breast, cervical, esophageal and pancreatic cancers and malignant melanoma^15,22,24,27,35–38^, but it is found to be activated in ovarian cancer and clear-cell renal carcinoma^18,25,39^. In skin cancers, PARD3 shows dual effects depending on the tumor type^16^. In terms of HCC, a study reported the association between overexpression of PARD3 and extrahepatic metastasis, and suggested one of its possible mechanisms. However, the specific role and detailed mechanism of PARD3 in HCC has not been fully elucidated^17^. Hence, we performed the bioinformatic analysis using several independent databases to explore the potential functions of PARD3 in HCC, including pathway interactions, immune infiltration, and long-term survival.

Based on TCGA database, PARD3 expressed differentially in 24 types of cancers (Fig. 1A). Thereinto, the expression levels in two types of cancers were inconsistent with previous studies, including lung cancer and pancreatic cancer^22,27^, which may derive in part from data collection approaches and patients’ biological properties. More specifically, our study provided direct evidences that PARD3 was an independent risk factor for HCC development. Firstly, PARD3 expression was dramatically higher in HCC than in normal liver tissues (Fig. 1B–I). Secondly, high PARD3 expression group contained more patients with advanced pathologic stages, vascular invasion, and TP53 Mut, suggesting that overexpression of PARD3 was significantly associated with poor clinicopathologic features with a good predictive power (AUC = 0.835, Fig. 2 and Table 1). Thirdly, elevated PARD3 expression led to shorter OS and DSS in both whites and Asians regardless of gender, age and Child–Pugh grade (Fig. 3A,B). All these results proved PARD3 as a potential prognostic biomarker of HCC.

Thus, we further explored the possible mechanism by which high PARD3 expression worsens the outcomes of HCC patients. As an adverse prognostic indicator of HCC, PARD3 was involved in many pivotal mechanisms in cancer, including cell cycle^40^, DNA damage and repair^41^, and cell motility^42^ (Table 2). It is well known that genomic instability and mutagenesis, which caused by erroneous DNA repair, are closely correlated with poor prognosis and drug resistance in HCC^41^. TP53 is universally recognized as a hub gene in responding to DNA damage and guarding the genome, and its mutation is observed in about half of all solid tumors, including HCC^43,44^. Furthermore, p38MAPK is able to control p53 activation via direct phosphorylation. Based on our result of enrichment analysis that PARD3 was associated with MAPK pathway and TP53 regulation (Table 2), we hypothesized PARD3 may affect the formation and progression of HCC by regulating TP53 via MAPK pathway. However, the hypothesis requires further investigation. Rho Family GTPases, which is closely interact with PARD3, were widely reported to regulate cell cycle and cell motility across human cancer of different origins^42^. PARD3 directly activates Rac1, promoting proliferation and motility of cancer cells, and leads to tumorigenesis, angiogenesis, invasion and metastasis^45–47^. Likewise, Par complex also links Rho small GTPases to regulate asymmetrical cell division and cell polarization^48^, which manipulate EMT and mesenchymal-epithelial transition (MET)^49^. Our findings that PARD3 was significantly implicated in Rho pathway also provided evidences to confirm this theory (Table 2). In addition, some lncRNAs showed significant correlations with PARD3 expression, such as FAM83A-AS1, which is involved in HCC (Table 2)^50^.

Remarkably, we found that PARD3 and its associated DEGs mainly participated in cellular response to metal ions (Fig. 4C,D, and Table 2). Previous studies have provided a possible relationship between metal ion homeostasis and vascular invasion in HCC, which may be mediated by p53^51–53^. Disturbance in Cu and Zn homeostasis has been reported as a significant factor associated with tumor proliferation, angiogenesis and invasion in HCC, and furthermore, cellular response to Cu and Zn is probably involved in mitochondrial accumulation and stability of p53, so as to influence proliferation and apoptosis of hepatoma cells^54–56^.

In the past decades, reprogramming of energy metabolism was added into the list of cancer hallmarks^29^. Interestingly, we found many co-expression genes and pathways related to PARD3 were involved in deregulation of metabolisms, covering types of metabolic processes like fat acids, amino acids and pyrimidines (Table 2). In order to sustain prodigious proliferation, tumors exert a specialized metabolism that differs from normal tissues. During the period, tumors recruit abundant nucleotides to maintain unlimited replicative potential, and uptake more nutrients to support unchecked cell growth. In particular, alterations in metabolism fatty acid and glycine, serine and threonine have been investigated as a promoter of HCC initiation and progression^57,58^. Moreover, SLC22A1, a DEG inversely related to PARD3 (Fig. 4E), is a key regulator of metabolism, which is extensively considered as a suppressor of HCC development^59–61^.

Besides, PPI enrichment analysis screened SAA1-related cluster and CYP-related cluster as the two most crucial MCODE subnetworks, both of which were involved in metabolism and homeostasis (Fig. 5). Recent study showed that downregulated SAA1 was closely associated with progression of HCC and low anti-tumor immune infiltrating^62^; and CYP families might impact HCC cell viability via modulating biotransformation^63^. The results provided supporting evidence that PARD3 might promote HCC via regulating metabolism and homeostasis.

Recently, cellular metabolism has emerged as a determinant of the viability and function of both tumor cells and immune cells. Meanwhile, tumor metabolism is reported as an immune checkpoint^58,64^. As discussed above, PARD3 was linked to some important metabolic processes, and meanwhile, several enriched pathways were also associated with immune response (Table S4). Thus, we hypothesized that there may be an association between PARD3 and immune infiltration. As expected, PARD3 is correlated with diverse immune infiltration levels in HCC, especially DCs, cytotoxic cells and pDCs (Fig. 6). DCs are a heterogeneous population of professional antigen-presenting cells central to the induction and maintenance of adaptive immunity within tumor microenvironment^58,65^. In particular, two subsets of DCs exert the most potent antitumor functions, including conventional DCs type 1 (cDC1s) that stimulate T cell proliferation, and pDCs that produce interferon-α (IFN-α)^65,66^. cDC1s not only take up and cross-present tumor antigens via major histocompatibility complex (MHC) class I to activate naive CD8^+^ T cells; but also support the cytotoxicity of CD8^+^ T cells by secreting large amounts of interleukin-12 (IL-12). Then, activated cytotoxic CD8^+^ T cells migrate to tumors and kill them^65^. pCDs play two opposite roles in tumor immunity depending on their subsets via inducing Treg or activating cytotoxic T cells respectively^65,66^. Based on our result that PARD3 negatively correlated with DCs and cytotoxic T cells, we speculate that immune infiltration related to PARD3 may contribute to the unfavorable outcomes for HCC, yet the specific regulation mechanism needed to further elucidate.

In summary, our study reveals that overexpression of PARD3 correlates with poor clinicopathologic features and adverse outcomes in HCC. Moreover, the crosstalk of cellular response of metal ion, metabolism and immune infiltration within tumor microenvironment may partly explain the function of PARD3 in HCC development. However, bioinformatic analysis based on TCGA also has some limitations. First, the sample sizes of blacks and stage IV in LIHC may be too small to show a significant difference between groups. Additionally, transcriptome sequencing cannot directly reflect the protein activity and expression level. Therefore, our results should be verified by further research using sufficient HCC clinical samples, and detailed mechanisms of PARD3 need investigating more intensively. Despite the limitations, our findings provide multilevel evidence for the value of PARD3 as a potential prognostic biomarker and therapeutic target of HCC.

## Materials and methods

### RNA sequencing data and processing

RNA-Seq data (Workflow Type: HTSeq-FPKM) and corresponding clinical information were retrieved from TCGA-LIHC project, among which 371 HCC patients with complete survival information were retained. Then, level 3 HTseq-FPKM data were transformed to transcripts per million reads (TPM) for further analysis. Unavailable or unknown clinical data were treated as missing values^67^. RNA-Seq data of multiple cancer types were downloaded from the online database UCSC Xena (https://xenabrowser.net/datapages/), and analyzed using Toil^68^. This study complied with the publication guidelines provided by TCGA.

### Differentially expressed gene analysis

DESeq2 package was used to identify DEGs^69^. The cut-off value of PARD3 expression was determined by its median value, and the thresholds were defined as |log fold change (log FC)| > 2 and adjusted *p*-value < 0.05.

The differential expression of PARD3 was simultaneously validated using Gene Expression Omnibus (GEO) database (https://www.ncbi.nlm.nih.gov/geo), including three independent cohorts (GSE14520, GSE76427 and GSE121248)^70–72^.

### Functional enrichment analysis

#### Metascape analysis

Metascape (http://metascape.org) was used as a gene list analysis tool to conduct GO enrichment analysis of DEGs^73^, including BPs, MFs and CCs. *P*-value < 0.05, minimum count > 3 and enrichment factor > 1.5 were considered to be significant. The Cytoscape plug-in MCODE was used to screen crucial clustering subnetworks in PPI network.

#### Gene set enrichment analysis

GSEA was used for Kyoto Encyclopedia of Genes and Genomes (KEGG) pathway enrichment, which was performed with 1000 permutations for each analysis using curated gene sets (C2.cp.v7.0.symbols.gmt) as the reference gene set^74^. Visualization and statistics were carried out by R package clusterProfiler^75^. Adjusted *p*-value < 0.05, false discovery rate (FDR) *q*-value < 0.25 and |normalized enrichment score (NES)| > 1 were considered to be significant.

#### Immune infiltration analysis

The relative abundance of each immunocyte type was described with EC in single-sample Gene Set Enrichment Analysis (ssGSEA). ECs for 24 types of TIICs were quantified using GSVA package in R as reported previously^76^.

The immune infiltration analysis of PARD3 was replicated using TISIDB database (http://cis.hku.hk/TISIDB)^77^.

### Prognostic model generation and statistical analysis

All statistics were performed using R (v.3.6.2). The PARD3 expression levels between tumor and normal tissues in paired or non-paired samples were compared using Wilcoxon signed-rank test and Wilcoxon rank sum test, respectively. The discrimination ability of PARD3 in HCC was evaluated using the AUC in ROC^78^. The correlation between PARD3 expression and screened DEGs were analyzed using Spearman’s correlation. The correlation between PARD3 expression and immune infiltration were analyzed using Spearman’s correlation, while ECs of the two groups with different expression level were compared using Wilcoxon rank test. The relationship between clinicopathological features and PARD3 expression in HCC patients was assessed using Kruskal–Wallis test, Wilcoxon rank test or Spearman’s correlation, while prognostic relevance of the two groups with different expression level were compared using Pearson χ^2^ test, Fisher exact test or univariate logistic regression. A survival curve was plotted using Kaplan–Meier method, and analyzed by Cox regression. Baseline variables with a p-value < 0.1 on univariate analysis were included in multivariate Cox regression model^79,80^. A nomogram was generated to predict the prognosis of HCC based on the result of multivariate Cox regression analysis, including significant clinical characteristics and PARD3 expression. C-index was used to validate the predictive power of the model^81^. Statistical results were displayed with *p*-value, and hazard ratio (HR) at a 95% confidence interval (95% CI). *p*-value < 0.05 were considered to be statistically significant.

## Supplementary Information

Supplementary Information 1.

Supplementary Information 2.

Supplementary Information 3.

Supplementary Information 4.

Supplementary Information 5.

Supplementary Information 6.

Supplementary Information 7.

Supplementary Information 8.

Supplementary Information 9.

Supplementary Information 10.

Supplementary Information 11.

Supplementary Information 12.

Supplementary Information 13.

## Data Availability

The datasets used and/or analyzed during the current study are available from the corresponding author on reasonable request.
