# Supplementary material for: High expression of PARD3 predicts poor prognosis in hepatocellular carcinoma
Source: Sci Rep. 2021 May 26;11:11078. doi: 10.1038/s41598-021-90507-w (PMC8154901; doi:10.1038/s41598-021-90507-w)
Supplement: Supplementary file 1 — Supplementary Information 1. [file 41598_2021_90507_MOESM1_ESM.docx]

High expression of PARD3 predicts poor prognosis in hepatocellular carcinoma

Songwei Li ^1^, Jian Huang ^2^, Fan Yang ^1^, Haiping Zeng ^3^, Yuyun Tong ^1^ and Kejia Li ^2,^*

^1^ Department of Interventional radiology, The Second Affiliated Hospital of Kunming Medical University, 374 Dianmian Avenue, Kunming, Yunnan 650101, China

^2^ Department of Pharmacy, The Second Affiliated Hospital of Kunming Medical University, 374 Dianmian Avenue, Kunming, Yunnan 650101, China

^3^ Department of Infection management, The Second Affiliated Hospital of Kunming Medical University, 374 Dianmian Avenue, Kunming, Yunnan 650101, China

***** Correspondence: [taxol2000@126.com](mailto:taxol2000@126.com)

**Supplementary files**

Supplementary file 1: Supplementary Information.

Supplementary file 2: Figure S1. OS validation.

Supplementary file 3: Figure S2. immune infiltration validation using TISIDB.

Supplementary file 4: Table S1. Cox regression (OS).

Supplementary file 5: Table S2. Cox regression (DSS).

Supplementary file 6: Table S3. Co-expression genes.

Supplementary file 7: Table S4. GSEA pathway enrichment.

Supplementary file 8 (raw data): GSE14520_series_matrix

Supplementary file 9 (raw data): GSE76427_series_matrix

Supplementary file 10 (raw data): GSE121248_series_matrix

Supplementary file 11 (raw data): TCGA-clinical

Supplementary file 12 (raw data): TCGA-exposure

Supplementary file 13 (raw data): TCGA-mRNA expression
