# Supplementary figures and images for: High expression of PARD3 predicts poor prognosis in hepatocellular carcinoma
Source: Sci Rep. 2021 May 26;11:11078. doi: 10.1038/s41598-021-90507-w (PMC8154901; doi:10.1038/s41598-021-90507-w)

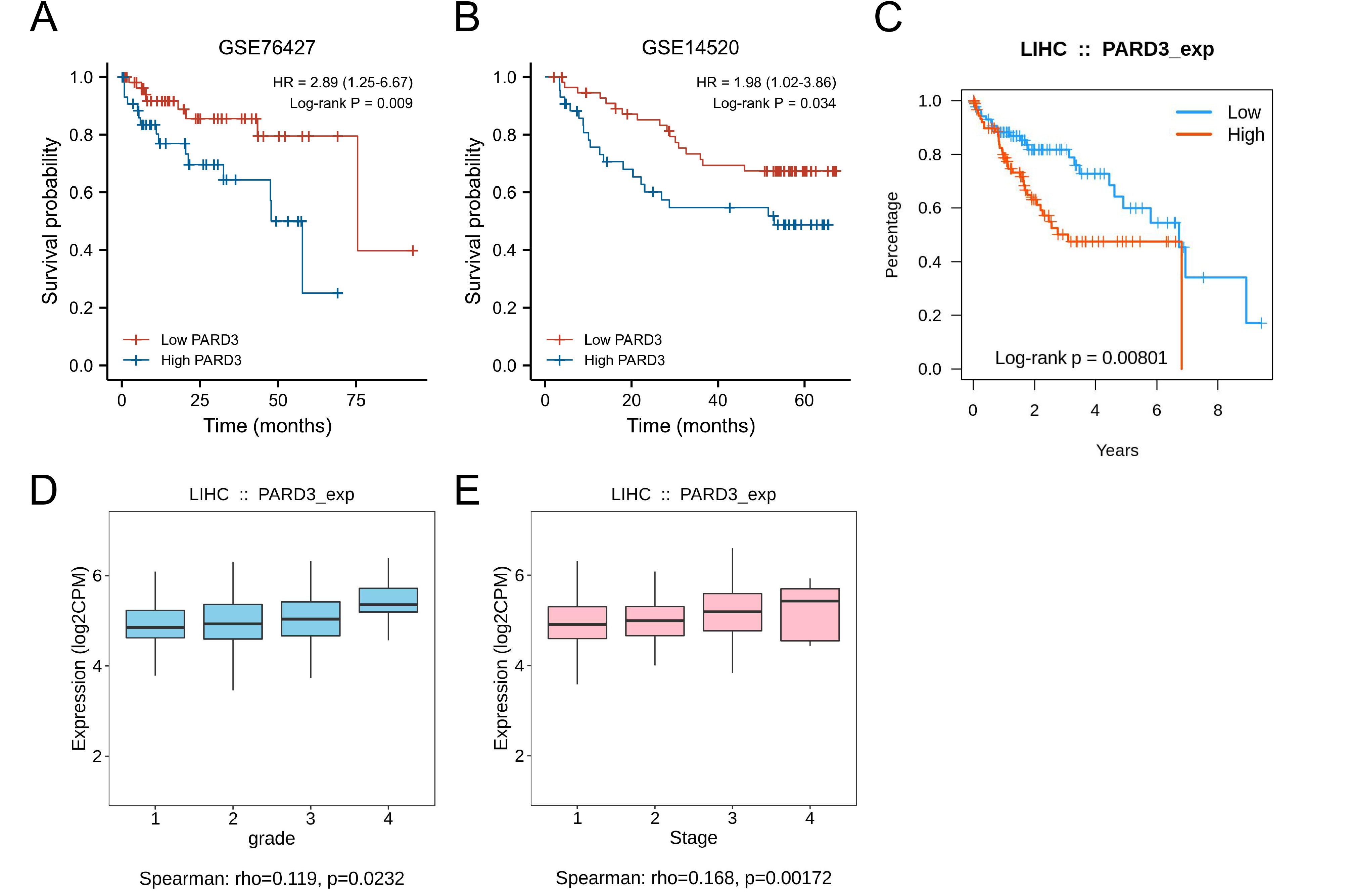

Supplement: Supplementary file 2 — Supplementary Information 2. [file 41598_2021_90507_MOESM2_ESM.tiff]

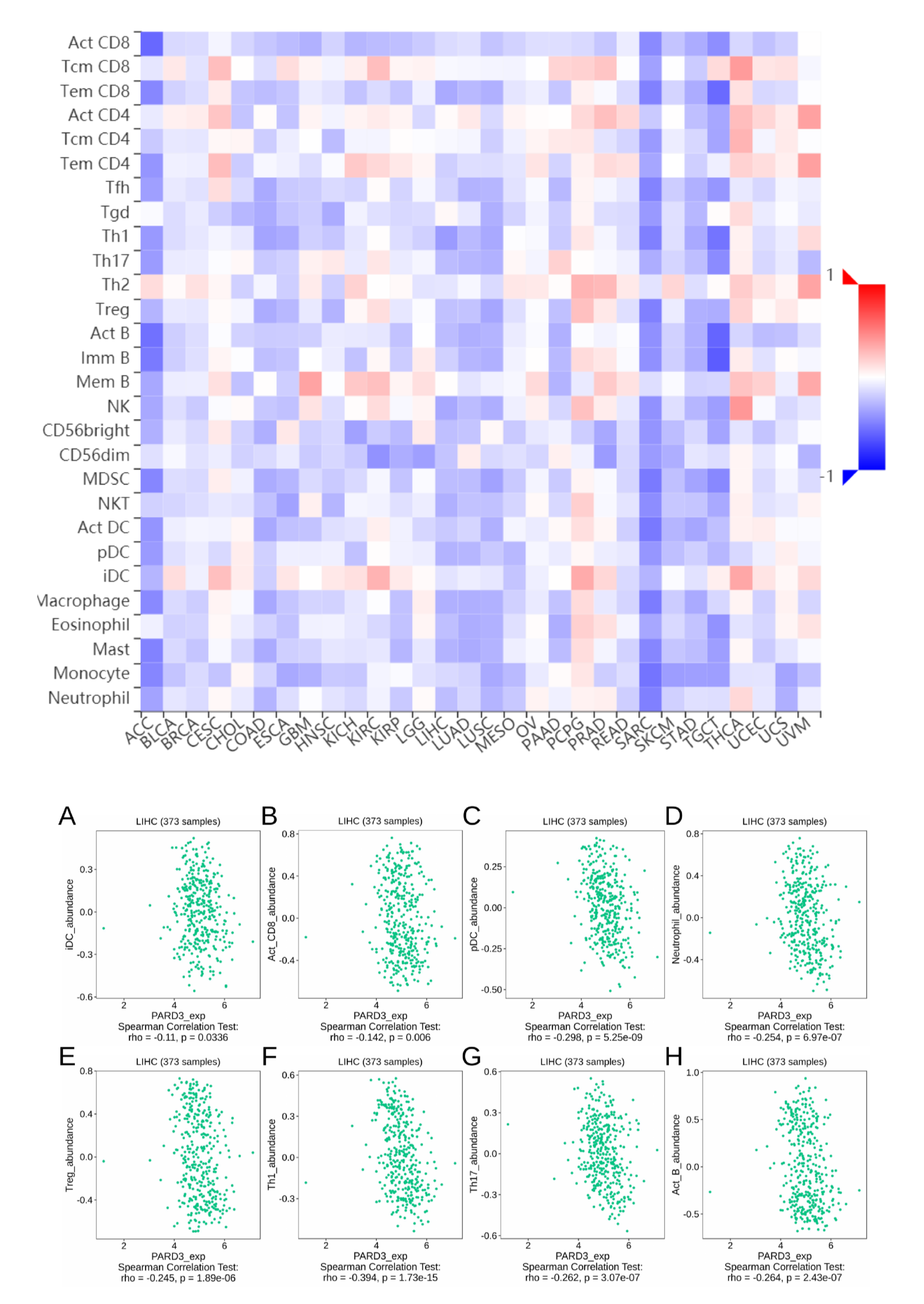

Supplement: Supplementary file 3 — Supplementary Information 3. [file 41598_2021_90507_MOESM3_ESM.tif]
